# Diet Composition, Adherence to Calorie Restriction, and Cardiometabolic Disease Risk Modification

**DOI:** 10.1093/geroni/igaa057.3104

**Published:** 2020-12-16

**Authors:** Sai Krupa Das, Alistair Senior, Rachel Silver, Cheryl Gilhooly, David Le Couteur

**Affiliations:** 1 Tufts University, Boston, Massachusetts, United States; 2 University of Sydney, Camperdown, New South Wales, Australia; 3 Jean Mayer USDA Human Nutrition Research Center on Aging at Tufts University, Boston, Massachusetts, United States

## Abstract

Calorie restriction (CR) is a promising strategy to attenuate age-related disease risk. Higher protein diets enhance satiety but may also impair metabolic health and accelerate aging. The effect of higher protein intake on adherence to CR and cardiometabolic markers of healthspan remains unknown. We used the Geometric Framework for Nutrition to examine the association between diet composition and 1) CR adherence; and 2) cardiometabolic risk factors during a 2-year intervention. The CR group consumed higher percentage energy from protein and lower fat at 12 months compared to baseline (logit % protein=0.1; 95% CI=0.05, 0.15; logit % fat= -0.12; CI=-0.29, -0.18). Higher protein intake over the 2-year intervention was associated with higher adherence to CR. No effect of diet composition on cardiometabolic risk factors was observed. These findings suggest that dietary protein plays a critical role in adherence to CR with no adverse effects on cardiometabolic markers of healthspan. Part of a symposium sponsored by the Nutrition Interest Group.

